# Gut microbiota in alcohol-related liver disease: pathophysiology and gut-brain cross talk

**DOI:** 10.3389/fphar.2023.1258062

**Published:** 2023-08-04

**Authors:** Lin Zhu, Yixuan Wang, Calvin Q. Pan, Huichun Xing

**Affiliations:** ^1^ Center of Liver Diseases Division 3, Beijing Ditan Hospital, Capital Medical University, Beijing, China; ^2^ Division of Gastroenterology and Hepatology, BaoJi Central Hospital, Shaanxi, China; ^3^ Division of Gastroenterology and Hepatology, NYU Langone Health, New York University School of Medicine, New York, NY, United States; ^4^ Center of Liver Diseases, Peking University Ditan Teaching Hospital, Beijing, China

**Keywords:** alcohol-related liver disease, alcohol-related cirrhosis, alcohol-related hepatitis, alcohol use disorder, gut dysbiosis

## Abstract

Alcohol-related liver disease (ALD) from excessive alcohol intake has a unique gut microbiota profile. The disease progression-free survival in ALD patients has been associated with the degree of gut dysbiosis. The vicious cycles between gut dysbiosis and the disease progression in ALD including: an increase of acetaldehyde production and bile acid secretion, impaired gut barrier, enrichment of circulating microbiota, toxicities of microbiota metabolites, a cascade of pro-inflammatory chemokines or cytokines, and augmentation in the generation of reactive oxygen species. The aforementioned pathophysiology process plays an important role in different disease stages with a spectrum of alcohol hepatitis, ALD cirrhosis, neurological dysfunction, and hepatocellular carcinoma. This review aims to illustrate the pathophysiology of gut microbiota and clarify the gut-brain crosstalk in ALD, which may provide the opportunity of identifying target points for future therapeutic intervention in ALD.

## 1 Introduction

Alcohol-related liver disease (ALD) has become an important public health problem with high incidence and mortality (Llopis et al., 2016; EASL Clinical Practice Guidelines: European Association for the Study of the Liver, 2018). It has a variety of clinical presentations with a spectrum from alcoholic hepatitis to liver cirrhosis. ALD has become a worldwide public health concern (approximately 4.2% of the population) and caused approximately 5.9% of all deaths every year (2018). However, 2.3 billion people between the ages of 15 and 59 were still active alcohol drinkers (Borrelli et al., 2022). The economic burden of ALD contributes significantly to the healthcare cost, which has been estimated at 125 billion Euros in the European Union and 249 billion dollars in the United States in 2010 (Axley et al., 2019).

The gut microbiome thrives in our alimentary tract in a symbiotic manner and affects the function of the gastrointestinal tract or liver, and *vice versa* (Gupta et al., 2021). In the last decade, lots of articles illustrated the pathophysiology of ALD and gut microbiota (Mutlu et al., 2012; Leclercq et al., 2014b; Bajaj et al., 2017; Brandl et al., 2018; Ciocan et al., 2018; Chu et al., 2020; Lang et al., 2020; Bajaj et al., 2021). To be specific, the gut microbiota contributed to individual susceptibility to ALD through the metabolic pathway, immune signaling pathway, and gut-brain axis (Queipo-Ortuño et al., 2012; Llopis et al., 2016; Bajaj, 2019). Moreover, alcohol dependence and liver dysfunction have a negative impact on the gut microbiota in ALD patients (Dubinkina et al., 2017). This current review mainly focused on the pathophysiology of gut microbiota and clarifies the gut-brain cross talk in ALD, which may provide the opportunity of identifying target points for future therapeutic intervention in ALD. We believe that the review will enhance our understanding of the topic and provide clear information for the potential therapeutic intervention through the targeting therapy of gut microbiota.

## 2 Literature search method

The literature search was performed on the PubMed database for studies published between 1/2010 to 6/2022 by using the search strategy shown in the Supplementary Material S1. The current review included data from randomized controlled trials, observational cohort studies, and animal studies. Studies were excluded: 1) All reviews including meta-analysis or the sample size too small in the ALD arm (n < 10); 2) Patients or animals co-infected with other liver diseases. 3) Not related to our topic. Based on the keyword search on the aforementioned, a total of 1398 articles were identified, and 69 articles were finally enrolled (Supplementary Material S2). The literature search was performed by 2 authors (LZ and YW). The disagreement on the study selections was arbitrated by the discussions with the corresponding author (HX and CP) and resolved with the group consensus.

## 3 Alcohol effects on gut pathophysiology and microbiota

Alcohol has a direct toxic effect on endoplasmic reticulum structure and function in hepatocytes, also resulting in intestinal stem cell dysregulation and long-lasting intestinal damage (Howarth et al., 2012; Lu et al., 2017). Chronic alcohol intake also enhances Tumor Necrosis Factor (TNF)-α expression in the jejunum of mice, human intestinal monocytes, and macrophages (Chen et al., 2015a). Elevated systemic TNF-α contributed to the activation of TNF receptor I on intestinal epithelial cells and phosphorylation of myosin light chain kinase to redistribute tight junction proteins and increases intestinal permeability (Chen et al., 2015a). Additionally, alcohol-induced gut leakiness from binging alcohol has been linked to the increase of cytochrome P450-2E1(CYP2E1), apoptosis of enterocytes, and production of nitration followed by ubiquitin-dependent proteolytic degradation of the junctional complex proteins (Abdelmegeed et al., 2013). Alcohol suppressed antimicrobial-regenerating islet-derived (REG)-3B lectins and REG3G gene and protein expression. REG3B and REG3G, as secreted C-type lectins mainly expressed in the intestinal epithelial and Paneth cells, had bactericidal activity against Gram-positive and Gram-negative bacteria respectively (Wang et al., 2016). Moreover, alcohol feeding of mice also disrupted the gut mucus layer and diminished mucosal thickness (Grander et al., 2018). Several studies suggest that intestinal aerobes and facultative anaerobes, like *Ruminococcus*, *Prevotella*, *Collinsella*, *Staphylococcus*, *Corynebacterium, Escherichia*, *Streptococcus*, were responsible for alcohol dehydrogenase-mediated ethanol oxidation under aerobic and even microaerobic conditions (Suen et al., 2011; Tsuruya et al., 2016). The accumulation of acetaldehyde further inhibited the expression of tight junction protein and promotes mucosa-associated microbiota translocated through the intestinal barrier to mesenteric lymph nodes and liver (Wang et al., 2016).

Alcohol can also affect gut bacteria profiles and features directly or change the composition of gut biofilms through bile acid synthesis indirectly. In the presence of alcohol, pathogenic bacteria like A*cinetobacter baumannii* enhanced its virulence significantly correlates with the acidification of bacterial cultures (Nwugo et al., 2012). Chronic alcohol abuse suppressed the bacterial genes involved in the biosynthesis of saturated long-chain fatty acids (LCFAs), inhibiting the proliferation of *Lactobacilli* which metabolizes saturated LCFA (Chen et al., 2015b). Primary bile acids such as cholic acid and chenodeoxycholic acid were synthesized through the Cholesterol 7-alpha-hydroxycholesterol (CYP7A1) classical pathway or the Cholesterol 27-hydroxycholesterol (CYP27A1) alternative pathway (Chiang, 2013). Alcohol upregulated the expression of CYP7A1 and CYP27A1 via the activation of hepatic cannabinoid receptor type 1 and suppressed fibroblast growth factor 15 gene expression to promote the synthesis of bile acids (Xie et al., 2013). Continued alcohol misuse also induced human fibroblast growth factor 19 gene expression in biliary epithelial cells and ductular cells, followed by total and conjugated bile acids increased significantly (Bajaj et al., 2017; Brandl et al., 2018). Bile acids exert a bacteriostatic effect, directly damaging DNA and destroying the bacterial membrane of bacterial. (Merritt and Donaldson, 2009; Kakiyama et al., 2013).

## 4 Microbiota in patients with Alcohol-related liver disease

Alcoholics with dysbiosis had a reduced bacterial diversity, decreased abundance of *Bacteroidetes*, and increased *Proteobacteria* (Mutlu et al., 2012). The changes of gut microbiotas have been observed at both of the phylum and family levels in patients with alcohol dependence syndrome (ADS) (Leclercq et al., 2014b; Dubinkina et al., 2017). At the phylum level, *Bacteroidetes* and *Firmicutes* of alcoholics decreased significantly, whereas *Proteobacteria*, *Fusobacteria*, and *Actinobacteria* increased. At the family level, there was a decrease in *Ruminococcaceae* significantly but an increase in both *Lachnospiraceae* and *Enterobacteriaceae* (Leclercq et al., 2014b; Dubinkina et al., 2017). Also observed that *Prevotellaceae* was enriched in alcohol-related cirrhosis (ALC) compared with controls and other etiologies cirrhotics (Chen et al., 2011). Moreover, Bajaj has summarized articles about alcoholic cirrhosis and microbiota composition or function in humans from 2012 to 2018 (Bajaj, 2019). There were 2 updated novel-related studies from 2018 to now. It is shown that ALD patients who had a lower fungal diversity with an overgrowth of *Candida* and higher serum anti-Saccharomyces cerevisiae antibodies had increased mortality (Lang et al., 2020).

Upregulation of systemic inflammation including inflammation in the oral cavity, gut, and liver is postulated to be a motivating factor of ALD (Acharya et al., 2017). The oral mucosa serves as the first line of defense due to the function of salivary immunoglobulins, agglutinins, histatins, and lysozyme. Enrolled 102 cirrhotics (38% alcohol-related) to analyze salivary and stool microbiomes. They observed that the lower salivary microbiota ratio (calculated by *Lachnospiraceae* + *Ruminococcaceae* + *Clostridiales Incertae Sedis XIV*/*Streptococcaceae*), the lower cirrhosis dysbiosis ratio (calculated by *Lachnospiraceae* + R*uminococcaceae* + C*lostridiales Incertae Sedis XIV* + V*eillonelllaceae*/*Enterobacteriaceae* + *Bacteroidaceae*), indicates oral dysbiosis and gut dysbiosis respectively (Bajaj et al., 2015). Furthermore, patients marked with salivary dysbiosis confront a higher 90-day liver-related hospitalization rate than those not. The possible cause may be related to oral microbiota invading the gut under the changes in gut pH or bile acid dysregulation of ALD. Patients with ADS had significantly increased oral-oriented microbes like *Lactobacillus salivarius*, *Veillonella parvula*, and *Streptococcus salivarius* (Dubinkina et al., 2017). When applying periodontal therapy in alcohol-related cirrhotics, the model for end-stage liver disease (MELD) scores were improved as the results of alleviating oral-gut dysbiosis, and decreased endotoxin, lipopolysaccharide-binding protein (Bajaj et al., 2018).

## 5 Metabolites from microbiota in patients with Alcohol-related liver disease

Gut microbe-derived metabolites mainly included long-chain fatty acids (LCFAs), short-chain fatty acids (SCFAs), mucus, secondary bile acids, indole or phenol derivatives, and vitamin B (Table 1) (Fan and Pedersen, 2021). Methods like integrated analyses of the microbiome and linked metabolomes as well as microbiome-wide association studies can be used to profile the gut metabolome characteristic, including shotgun-based sequencing, various bioinformatic algorithms, strain-level profiling, and so on (Fan and Pedersen, 2021). Bioinformatics analyses such as the Kyoto Encyclopedia of Genes and Genomes (KEGG) analysis have been applied to identify potential mechanistic links between the gut microbiome and metabolome. KEGG is a database and helpful tool for understanding most of the known metabolic pathways based on sequence similarity to proteins with known functional characteristics. For ALD patients, seven KEGG pathways were significantly increased (Galactose metabolism Porphyrin, chlorophyll metabolism, ABC transporters, phosphotransferase system, fructose or mannose metabolism, glutathione metabolism, and biosynthesis of siderophore group non-ribosomal peptides) (Dubinkina et al., 2017). Besides, there were significantly lower fecal metabolites focused on bioenergetics (citrate, malate, or phosphate), amino acids (threonine, ornithine, or serine), and pyrimidine intermediates (ribosine, orotic acid, or hexonate) (Kakiyama et al., 2013; Bajaj et al., 2017; Brandl et al., 2018; Ciocan et al., 2018; Fan and Pedersen, 2021).

**TABLE 1:** Gut metabolites as protective factors in ALD.

Microbiota metabolites		Reference	Trials type	Key findings
Bile acids		Ciocan et al. (2018)	Human studies	Bile acids positively correlates with the histological severity of liver inflammation via the activation of various nuclear receptors, such as FXR.
		Llopis et al. (2016)	Animal model	CDCA and UDCA of mice were decreased with the progression form non- hepatitis to sAH.
		Kakiyama et al. (2014)	Human studies	In ALC patients, the conversion of primary BAs to secondary BAs decreased
Arachidonic acid		Miyamoto et al. (2019)	Animal model	Associated with a low level of VLDL and a high level of HDL.
		Zheng et al. (2020)	Animal model	*Lactobacillus* were involved in arachidonic acid metabolism and reverse arachidonic acid-mediated adipose inflammation and liver lipid accumulation
Butyrate		Cresci et al. (2017)	Animal model	Protecting the gut barrier in preserving tight junction protein expression
		Roychowdhury et al. (2019)	Animal model	Depletion of butyrate producing bacteria is associated with increased endotoxemia, hepatic inflammation, steatosis, and gut permeability
Tryptophan	IAA	Shu et al. (2015)	Cell model	*Acinetobacter baumannii* can degrade IAA.
	IPA	Dodd et al. (2017)	Cell model	IPA is produced by gut symbiont *Clostridium sporogenes*
			+Animal model	
		Jennis et al. (2018)	Animal model	Regulate intestinal barrier function and decrease intestinal permeability mediated by the pregnane X receptor

### 5.1 Bile acids involved in the disease progression

Gut microbiota played a key role in bile acid homeostasis. Several studies found that alcohol-related hepatitis or cirrhosis in patients with ALD had higher total fecal bile acids, total secondary bile acids, deoxycholic acid, lithocholic acid, and a higher concentration of serum conjugated deoxycholic acid (Kakiyama et al., 2014; Ciocan et al., 2018). As a choloylglycine hydrolase produced by gut microbiota, bile salt hydrolase (BSHs) can catalyze the hydrolysis of conjugated bile salts into deconjugated bile acids. As a result, the transformation of primary bile acids to secondary bile acids increased. BSHs were distributed in 591 intestinal bacterial strains within 117 genera in human microbiota, mainly distributed in the family *Firmicutes* and *Proteobacteria*, genus *Bacillus*, *Staphylococcus*, *Bacteroides*, *Lactobacillus*, *Enterococcus*, and *Clostridium* (Song et al., 2019). Gut microbiota, Serving as an important metabolic organ of the host (Chen et al., 2011), has significant effects on the metabolism of bile acids in patients with ALD. Bile acids positively correlate with the histological severity of liver inflammation via the activation of various nuclear receptors, such as the farnesoid X receptor (FXR) (Ciocan et al., 2018). In animal studies, under the same drinking state, the fecal chenodeoxycholic acid (CDCA) and ursodeoxycholic acid (UDCA) of mice were decreased with the progression from non-hepatitis to severe AH (sAH) (Llopis et al., 2016). Ursodeoxycholic acid (UDCA), a hydrophilic bile acid that is the hepatoprotective metabolite of CDCA could induce the production of class I alcohol dehydrogenase (ADH1) and accelerate the transformation and metabolism of ethanol. Alcohol dehydrogenase 1 (ADH1) gene polymorphism may be one of the reasons for the different susceptibility to ALD (Ehlers et al., 2012).

### 5.2 Other gut metabolites served as the protective factors in Alcohol-related liver disease

Some gut microbe-derived metabolites involving SCFAs, LCFAs, mucus, indole derivatives, and vitamin B had protective effects on the gut and liver (Miyamoto et al., 2019; Zheng et al., 2020). *Lactobacillus* was involved in arachidonic acid metabolism and can reverse arachidonic acid-mediated adipose inflammation and liver lipid accumulation, thus exerting protective effects on ALD liver steatosis (Miyamoto et al., 2019; Zheng et al., 2020). Moreover, arachidonic acid can be transformed into potent bioactive compounds like lipoxins, prostaglandin E1, and prostaglandin I2, exerting anti-inflammatory effects in ALD (Das, 2019). Butyrate, as the major fuel source for the colonocyte and one of three predominant SCFAs (acetate, propionate, and butyrate), has a protective effect on the gut barrier in preserving tight junction protein expression (Cresci et al., 2017; Roychowdhury et al., 2019). Thus, the butyrate-producing bacteria *Faecalibacterium prausnitzii*, a dominant member of the *Clostridium leptum* subgroup, possessed anti-inflammatory and mucosal protective properties to mitigate ethanol-induced gut-liver injury (Rios-Covian et al., 2015).

The mucus barrier integrity is the first line of the intestinal tract. *Akkermansia muciniphila*, a mucin-degrading anaerobic bacterium that resides in the gut mucus layer, can utilize mucins as carbon and nitrogen source to restore mucus thickening and control the host mucus turnover (Everard et al., 2013; Grander et al., 2018). Moreover, *Gegus Roseburia* isolated from the gut can recover gut mucus integrity through upregulation of the protein occludin and MUC2(Grander et al., 2018). Restoration of mucus barrier function decreased expression of pro-inflammatory cytokines and dissemination of endotoxins in ALD (Grander et al., 2018).

Diverse bacterial species have been reported to produce tryptophan catabolites (Hendrikx et al., 2019). Indole-3-acetic acid (IAA), as a tryptophan catabolite and a microbiota-derived ligand of the aryl hydrocarbon receptor (AHR) which regulates AhR-dependent IL22 and Reg3g expression, strengthened the integrity of intestinal mucosa and reduced alcoholic-related liver inflammation (Roager and Licht, 2018). IAA degradation by a common pathogenic pathogen of ALD, such as *Acinetobacter baumannii*, via the indole-3-pyruvate (IPyA) pathway, which primarily involves IAA catabolic sites, catechol-degrading genes, and phenylacetic acid degrading genes, may be linked to the significant reduction of IAA levels in feces of patients with alcoholic hepatitis (Shu et al., 2015; Lin et al., 2018). Indolepropionic acid (IPA) is another tryptophan catabolite produced by gut symbiont *Clostridium sporogenes*, a bacteria from the phylum *Firmicutes*. *Clostridium sporogenes* can generate aromatic amino acid (tryptophan, phenylalanine, and tyrosine) metabolites via aromatic amino acid aminotransferase reductive pathways (Dodd et al., 2017). IPA can regulate intestinal barrier function and decrease intestinal permeability mediated by the pregnane X receptor (Jennis et al., 2018). The vitamin B was able to regulate the methionine metabolic cycle in the liver and essential for the production of glutathione, the antioxidant against oxidative liver injury (Halsted, 2013). Some related species of *Bifidobacterium* and *Lactobacillus* (like *Bifidobacterium longum* and *Lactobacillus reuteri*), normally existing in the human gut, were involved in the *denovo* synthesis process of folate and vitamin B12. These species may have a vital role in the treatment of ALD (LeBlanc et al., 2013).

## 6 Gut microbiota and immune response

**FIGURE 1:**
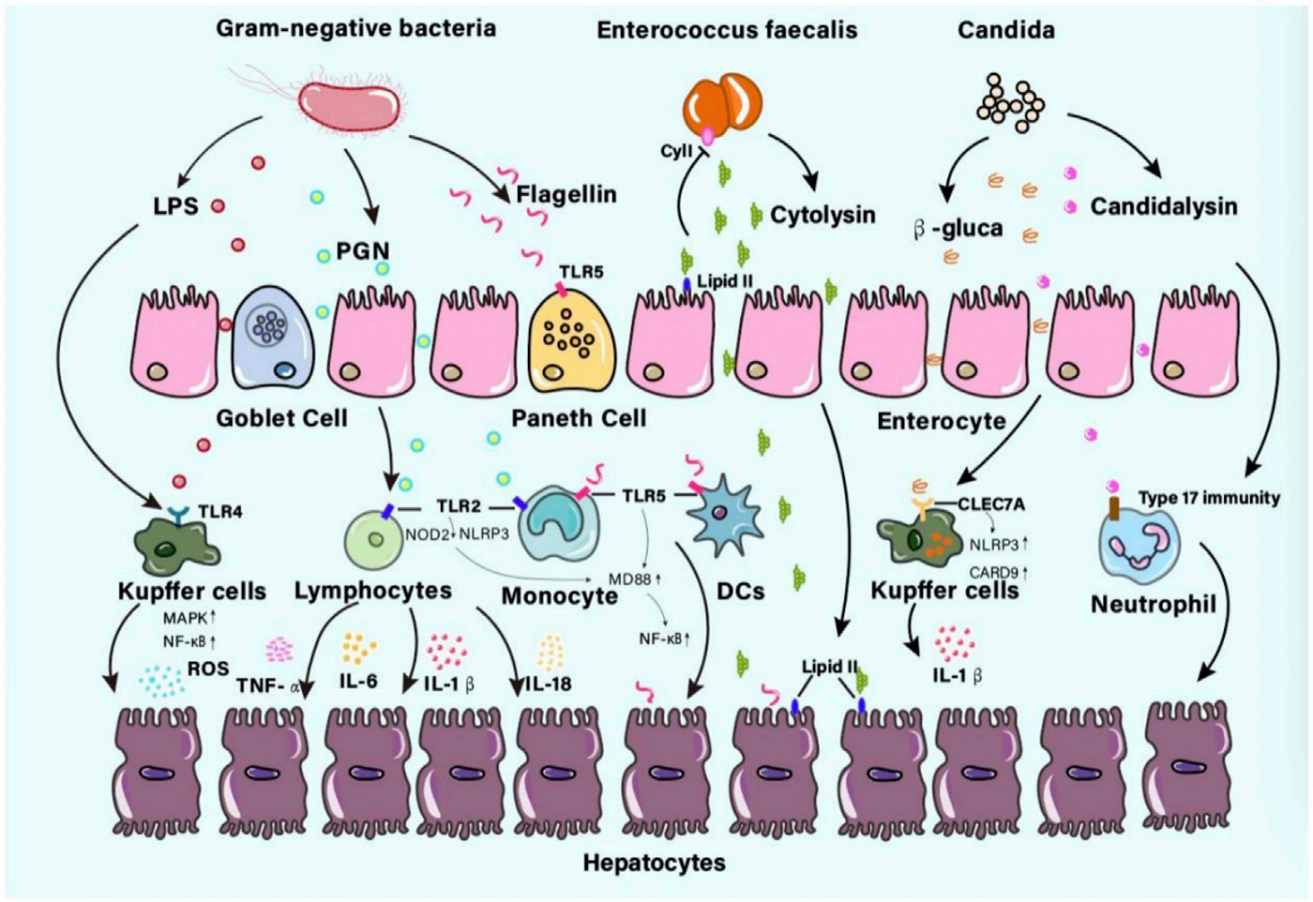
The pathogens or components interact with the liver in ALD patients Gut microbes can get across the intestinal barrier then interact with the liver. The pathogens or components like lipopolysaccharide (LPS), peptidoglycan (PGN), flagellin, cytolysin, β-glucan, or candidalysin, prolonged inflammatory immune response may contribute to fibrosis even cirrhosis. Abbreviations: LPS, lipopolysaccharide; PGN, peptidoglycan.

The appropriate immune response in the liver is dependent on the pattern recognition receptors expressed by liver sinusoidal endothelial cells, hepatocytes, Kupffer cells, dendritic cells, and liver-resident lymphocytes through the activation of receptors like Toll-like receptors (TLRs) (Jenne and Kubes, 2013). In ALD patients, gut microbiota can get across the intestinal barrier and circulate to the liver through the portal vein to interact with the liver (Bruellman and Llorente, 2021). As a result, a prolonged inflammatory immune response is activated, as shown in Figure 1.

### 6.1 Mechanism of gut microbiota aggravating liver injury

Recently, the pathophysiology of several bacteria or their products triggering liver immune injury has been studied extensively. Gram-negative bacteria were surrounded by a thin PGN cell wall. Muramyl dipeptide, the minimal bioactive cytosolic structure of PGN, can not only stimulate TLR2 receptors on lymphocytes and monocytes but also interact with Nod-like receptor proteins NOD2 or NLRP3 (Leclercq et al., 2014a). The MyD88-dependent pathway in lymphocytes or monocytes was activated, which lead to the production of TNF-α, IL-6, IL-1β, and IL-18 (Schroder and Tschopp, 2010). In a recent study on alcohol-dependence patients, there were pieces of evidence that PGN may also cross the gut barrier and contribute to the high concentration of PGN in the plasma (Leclercq et al., 2014a). As an antigen from Gram-negative bacteria surface, the LPS can bind into the pattern-recognition receptors such as TLR4 of the Kupffer cells in the liver and trigger the production of proinflammatory mediators including TNFα (Mukherjee et al., 2014; Llopis et al., 2016). Furthermore, this can induce the activation of mitogen-activated protein kinase and nuclear transcription factor kappa B(NF-κB) pathway and lead to reactive oxygen species and persistent liver inflammation in ALD (Mukherjee et al., 2014; Llopis et al., 2016). As a main component of the bacterial flagellum, flagellin plays an important role in bacterial motility. In an animal study, liver injury was observed in mice because of increased levels of flagellin (Yoon et al., 2012). The injury was thought to be an immune injury triggered by the activation of TLR5 in the antimicrobial peptide-secreting paneth cells, which had the unique protein-binding TLR for the flagellin component in b- and g-Proteobacteria. The activation of TLR5 further initiated the MyD88-dependent signaling pathway and promoted the proinflammatory transcription factor NF-κB, leading to immune clearance against flagellated bacteria (Yang and Yan, 2017). Since rapid immune responses to flagellin have been observed in hepatocytes, epithelial cells, monocytes, and DCs, and increased *Proteobacteria* were found in ALD-related studies, this has led to the disease progression in patients with ALD (Chen et al., 2011; Engen et al., 2015; Leclercq et al., 2020). Cytolysin is a two-subunit exotoxin secreted by *Enterococcus faecalis*. Cytolysin may use lipid II as a docking molecule to lyse target cell membranes such as bacteria, erythrocytes, or eukaryotic cells. However, it can prevent self-lysis through the activity of the immunity factor CyII and the pheromone quorum signaling pathway (Van Tyne et al., 2013). A study showed that alcoholic hepatitis patients had increased fecal numbers of *E. faecalis* (Duan et al., 2019), and the presence of cytolysin-positive (cytolytic) *E. faecalis* correlated with severe liver damage and high mortality in patients with ALD (Duan et al., 2019).

There are three predominant commensal fungal species in the human gut, which include *Candida*, *Saccharomyces cerevisiae*, and *Malassezia* (Wang et al., 2014). Alcohol-dependent patients displayed decreased gut fungal diversity and overgrowth of *Candida*. β-glucan is a cell wall polysaccharide found in most fungi like *Candida*. As a result of *candida* overgrowth, a higher concentration of β-glucan circulated to the liver and induced hepatic inflammation via a receptor of the C-type lectin domain family 7 member A (CLEC7A) on Kupffer cells (Yang et al., 2017). More specifically, the CLEC7A signaling pathway activates NLR family pyrin domain-containing 3 and caspase recruitment domain family member 9, leading to the production of mature IL-1β. IL-1β secretion contributed to hepatocyte injury and the development of ALD (Dambuza and Brown, 2015). Candidalysin is a cytolytic peptide toxin secreted by *Candida*, and associated with liver disease severity and mortality in alcohol-related hepatitis (AH) patients via participation in neutrophil recruitment and Type 17 immunity (Naglik et al., 2019).

### 6.2 Mechanism of gut microbiota attenuating liver injury

**FIGURE 2:**
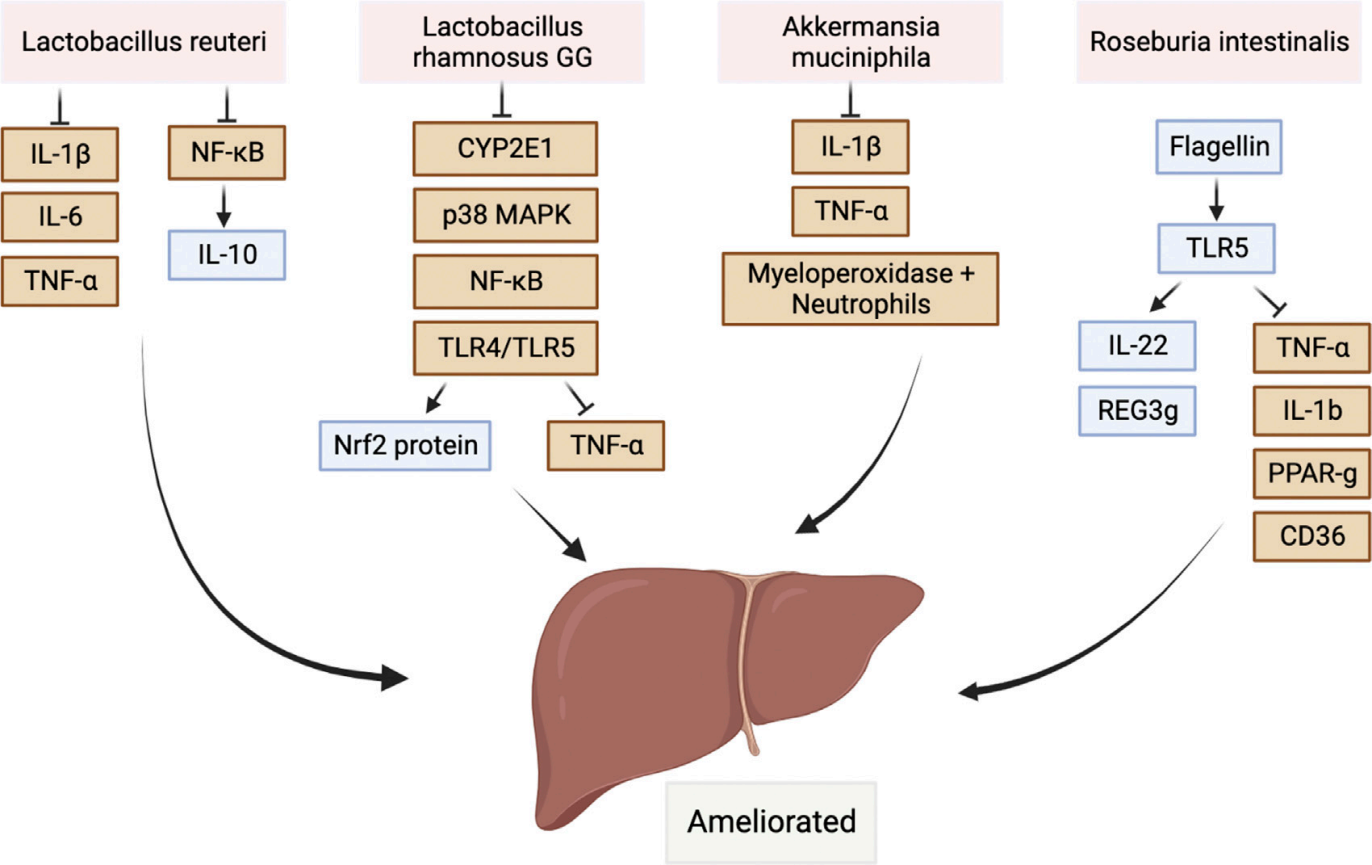
Protective commensal bacteria protect the liver by regulating the immune system. There were also some protective commensal bacteria such as *Lactobacillus*, Akkermansia muciniphila, and Roseburia can also protect the liver by regulating the immune system.Abbreviations: IL-1β, interleukin-1 beta; IL-6, interleukin-6; IL-10, interleukin-10; p38 MAPK, P38 mitogen-activated protein kinase; Nrf2 protein, nuclear factor erythroid 2-related factor 2 protein; REG3g, regenerating islet-derived protein 3 gamma; PPAR-g, Peroxisome proliferative activated receptor gamma; TLR, toll-like receptor; CD36, fatty acid transporter/CD36 molecule; NF-κB, nuclear transcription factor kappa B; CYP2E1, cytochrome P450-2E1.

In contrast, the attenuating effect on hepatic injury has been associated with several microbiota in the gut. Study showed that *L. reuteri* promoted the expression of the anti-inflammatory cytokine IL-10 by suppressing NF-κB signaling pathways (Hsu et al., 2017). They also decreased the expression levels of hepatic IL-1β, IL-6, and TNF-α leading to the reduction of necro-inflammation in hepatocytes (Hsu et al., 2017). Besides, *Lactobacillus rhamnosus GG* inhibited CYP2E1 expression, p38 MAP kinase phosphorylation, TLR4/TLR5, and NFκB activation, which upregulated Nrf2 protein levels and attenuated the production of proinflammatory TNFα in C57BL/6N mice of chronic liver injury from alcohol (Wang et al., 2013). In addition, the protective effects of *L. rhamnosus GG* in ALD were also documented. The mechanism was speculated as a process of up-regulating multiple inducers and stabilizers including intestinal trefoil factor for the mucus layer (Wang et al., 2013). Regarding the effects of *A. muciniphila*, the presence of the bacteria not only reduce IL-1β and TNF-α expression but also reduces infiltration of myeloperoxidase and neutrophils in ALD (Grander et al., 2018). In terms of restoring intestinal barrier function, it is worth noting that some butyrate-producing bacteria such as *Roseburia intestinalis* can possibly enhance the expression of IL-22 and REG3g through their flagellin binding with TLR5 and attenuated the hepatic expression of TNF-a and IL-1b as well as lipid transport factors (Seo et al., 2020), such as PPAR-g and CD36. Restoring intestinal barriers help ameliorate ALD (Seo et al., 2020). The protective effects were summarized in Figure 2. Flagellin is a double-edged sword that have some protective immune effects and the potential of inducing further liver injury, which depended on the bacterial density. Thus, caution should be taken when interpreting the study results or reviewing the contradictory data.

## 7 Gut-brain axis in Alcohol-related liver disease

The changes in gut microbiota also influence the function of the brain. The gut microbiota communicates with the brain via the neuroendocrine or neuroimmune mechanisms, known as the gut-brain axis. Gut microbiota can produce neurotransmitters, precursors like 5-hydroxytryptamine(5-HT), dopamine, noradrenaline, L-glutamic acid, γ-and aminobutyric acid (GABA), which can regulate the function of the central nervous system (Seo et al., 2020). In addition, other metabolites of gut microbiota, such as acetate, may affect the levels of 5-HT or other neurotransmitters and therefore influence human behavior (Holzer et al., 2012). Recently, Qamar et al., 2019 used the Ingenuity Pathway Analysis Software to identify the alteration in neurotransmitters associated with alcohol consumption.

Several bacteria such as *Streptococcus*, *Acinetobacter*, *Escherichia coli*, and *Clostridia* can produce 5-HT directly or generate it via a bacteria-mediated tryptophan metabolism pathway (Williams et al., 2014; Zhang et al., 2021). The 5-HT levels were always significantly increased in ALD patients when compared with those in non-ALD patients (Williams et al., 2014; Zhang et al., 2021). The activation of 5-HT receptors promoted dopamine release in the reward circuitry and increased the risk of alcohol addiction (Enoch et al., 2011). In addition, central noradrenergic neurons can convert dopamine to norepinephrine (Enoch et al., 2011). Norepinephrine can affect the growth of *Prevotella* and other anaerobic bacteria as it can increase virulence gene expression and enhance iron acquisition in *Clostridium* (Boyanova, 2017). Furthermore, several bacteria strains have been reported to be able to produce dopamine and norepinephrine. They included *E. coli*, *Proteus vulgaris*, *Staphylococcus aureus*, and *Bacillus subtilis* (Strandwitz, 2018). Noradrenergic neurons also can be regulated by transmitters like glutamate and GABA. The GABA was produced through the α-decarboxylation of glutamate by the enzyme glutamate decarboxylase (Sarasa et al., 2020). Chronic alcohol exposure increased the extracellular glutamate concentration and suppressed GABA activity, leading to decrease glutamatergic synaptic transmission and further alcohol consumption. However, acute alcohol exposure resulted in the opposite effects (Alasmari et al., 2018). As GABA-producing bacteria and neuroactive bacteria, *L. reuteri* and *L. rhamnosus*, act on intrinsic primary afferent neurons in the mouse model (Mao et al., 2013; Pokusaeva et al., 2017; Tsai et al., 2020; Zheng et al., 2020), which transmit microbial messages to the brain via the vagus nerve. As a result, alcohol-induced hepatitis was alleviated.

**FIGURE 3:**
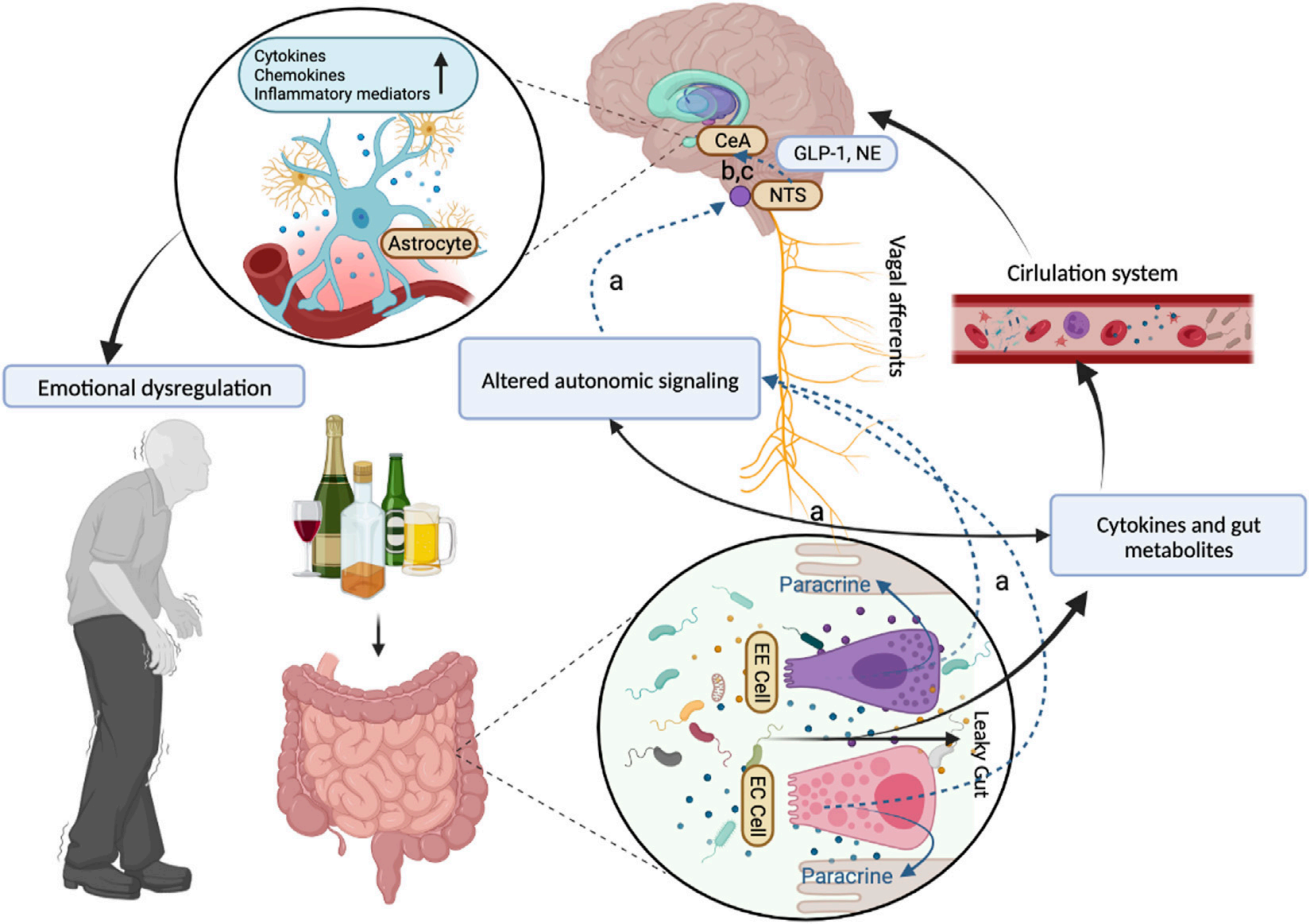
The role of the gut-brain cross talk in alcohol withdrawal reaction.The whole mechanism of associations between the gut-brain cross-talk and the mental and neurological abnormal manifestations in the alcohol withdrawal reaction. **(A)** The vagus afferent nerve senses change in relative bacterial abundance through two pathways: one route of communication may be through direct interactions between vagal afferent neurons and bacteria or their metabolic products. Another route includes the effects of paracrine signaling from enterochromaffin (EC) cells and from enteroendocrine (EE) cells that interact with the gut lumen causing secretion of factors that affect nearby cells in the gut wall. **(B)** The state of the gut microbiome is reported to the brain via vagal projections to the nucleus tractus solitarius (NTS) by changes in the activity of the vagus nerve and in the neurochemical phenotype of the vagal afferent neurons in the nodose ganglia and their targets in the NTS. The NTS in turn has viscerosensory projections to the central nucleus of the amygdala (CeA) that involves norepinephrine (NE). Neuroinflammation in regions like the central nucleus of the amygdala (CeA) and others can contribute to emotional dysregulation seen in withdrawal. **(C)** Neurons expressing glucagon-like peptide 1 (GLP-1) and glutamate have been described to participate in these NTS viscerosensory projections to the CeA. Abbreviations: EE Cell, enteroendocrine cells; EC Cell, enterochromaffin cells; NTS, nucleus tractus solitarius; CeA, central nucleus of the amygdala; GLP-1, glucagon-like peptide 1; NE, norepinephrine.

The anxiety/depression-like behaviors in patients with chronic alcohol intake have been associated with the increase (Zhao et al., 2020) of pathogenic colonies like *Actinobacteria* and *Adlercreutzia*, which drive changes in inflammatory signaling and cytokine release, leading to GABA-1 receptor changes in the prefrontal cortex and the brain-derived neurotrophic factor/α1 subunit of γ-aminobutyric acid A (Kelly et al., 2016; Xu et al., 2019), and finally influenced people’s emotions. For example, at the withdrawal affect stage, microbiome-immune disruptions have been identified as potential mediators of front-limbic anomalies and derived emotional dysregulation in the alcohol addiction cycle (Gorky and Schwaber, 2016). On the one hand, the “leaky gut” allows inflammatory molecules to leak outside of the gut into the blood to generate peripheral inflammation, further activating microglia and causing a neuroinflammatory response (Skinner et al., 2009; Keita and Söderholm, 2010). On the other hand, such alterations may prime the vagus nerve to alter its neuroprotective afferent signals or promote vagal signals that are in some harmful way (Gorky and Schwaber, 2016; Carbia et al., 2021). With the brain primed by vagal afferent alterations, it may be more susceptible to influence from the peripheral inflammatory response that occurs in response to alcohol withdrawal (Figure 3 and Suplementary Material S3). Another common brain disorder among ALD patients is dementia which mostly occurs in women and may triple the risk of death (Bahorik et al., 2021). Gut microbiota dysbiosis such as the increase of *Muribaculaceae* and the decrease of *Lactobacillus* can lead to the peripheral accumulation of phenylalanine and isoleucine, which stimulates the proliferation of pro-inflammatory T helper 1 cells, contributing to dementia-associated neuroinflammation (Sahlman et al., 2016). Gut-resident *Cyanobacteria*-generated neurotoxins involving β-N-methylamino-l-alanine and saxitoxin may further aggravate neurodegenerative disease (Alkasir et al., 2017).

Hepatic encephalopathy (HE) is one of the end-stage manifestations of ALD, characterized by disturbances in mental status and neurological function. In cirrhosis, ammonia crossed the blood-brain barrier and led to astrocyte swelling and reactive oxygen species formation (Alsahhar and Rahimi, 2019). The increased *Klebsiella*, *Proteus*, and *E. coli* were related to the increased plasma levels of ammonia and endotoxin, contributing to the occurrence of hepatic encephalopathy in ALD (Wang et al., 2019). Moreover, the gut microbiota diversity was decreased during acute episodes of overt HE, and the relative abundances of *Alistipes*, *Bacteroides*, and *Phascolarctobacterium* were respectively associated with HE recurrence and overall survival during the subsequent 1-year follow-up (Sung et al., 2019).

## 8 Conclusion

The gut microbiota dysbiosis in ALD was initiated by oral-gut dysbiosis, followed by gut microbes’ colonization and circulating microbiota change. The factors that persistently aggravate disease progression in ALD patients have been explored in recent studies, which included Alcohol intake, gut microbiota change, gut metabolic dysregulation, activated inflammatory immune response, and neurological or behavioral deficits, giving a better understanding of the pathophysiology of gut dysbiosis in ALD and the pathway of the gut-brain cross talk. Further investigations into the pathogenic of dysbiosis and the mechanisms of certain protective bacteria in ALD may provide the therapeutic targeting points for the treatment of ALD and aid in selecting effective regimens with a favorable safety profile.

## Abbreviations

AH, Alcohol-related Hepatitis; AHR, Aryl Hydrocarbon Receptor; ALD, Alcohol-related Liver Disease; BSHs, Bile Salt Hydrolase; CDCA, Chenodeoxycholic Acid; CYP2E1, Cytochrome P450-2E1; FXR,farnesoid X receptor; GABA, γ-Aminobutyric Acid; HE, Hepatic Encephalopathy; IAA, Indole-3-acetic Acid; IPA, Indolepropionic Acid; KEGG, Kyoto Encyclopedia of Genes and Genomes; LCFAs, Long-Chain Fatty Acids; LPS, Lipopolysaccharide; NF-κB, Nuclear Transcription Factor Kappa B; PGN, Peptidoglycan; REG3B, Antimicrobial-regener; TNF, Tumor Necrosis Factor; 5HT, 5-Hydroxytryptamine.
