# Supplementary material for: Gut microbiota in alcohol-related liver disease: pathophysiology and gut-brain cross talk
Source: Front Pharmacol. 2023 Aug 4;14:1258062. doi: 10.3389/fphar.2023.1258062 (PMC10436520; doi:10.3389/fphar.2023.1258062)
Supplement: Supplementary file 1 [file DataSheet1.docx]

**Online Supplementary Material**

This supplementary material has been provided by the authors to give readers additional information regarding their study.

Gut Microbiota in Alcoholic Liver Disease: Pathophysiology and Gut-Brain Cross Talk.

Short title: Gut Microbiota in Alcoholic Liver Disease

**Contents**

**Sup 1. Search Strategy** Page 2

**Sup 2. Table S1. Abstracts** Page 3

**Sup 3. Details of the gut-brain cross talk in alcohol withdrawal reaction** Page 9

**Search Strategy**

**PubMed, from Jan 01, 2010, to Jun 30, 2022 (1398 Articles)**

**Searching terms：**

1. ("Alcohol, Ethanol"[Mesh]) OR (Alcohol-Related Liver Disease) [Title/Abstract])

2. ("Microbiota, Alcohol"[Mesh]) OR ((Microbiota, Alcohol-Related Liver Disease [Title/Abstract]) OR (Microbiota, Alcohol Liver Disease [Title/Abstract]))

3. ("Dysbiosis, Alcohol"[Mesh]) OR ((Dysbiosis, Alcohol-Related Liver Disease [Title/Abstract]) OR (Dysbiosis, Alcohol Liver Disease [Title/Abstract]))

4. ("Microbiome, Alcohol"[Mesh]) OR ((Microbiome, Alcohol-Related Liver Disease [Title/Abstract]) OR (Microbiome, Alcohol Liver Disease [Title/Abstract]))

5. ("Hepatic Encephalopathy"[Mesh]) OR (Hepatic Encephalopathy, Alcohol Liver Disease [Title/Abstract]) OR (Hepatic Encephalopathy, Alcohol, Liver [Title/Abstract]))

1. (Immunity, Alcohol, Liver [Title/Abstract]) OR (Immune Dysfunction, Alcohol, Liver [Title/Abstract]) OR (Immunology, Alcohol, Liver [Title/Abstract])
2. ((Inflammation, Alcohol, Liver [Title/Abstract]) OR (inflammatory, Alcohol, Liver [Title/Abstract])
3. (Fungus, Gut, Alcohol [Title/Abstract]) OR (Bacteria, Gut, Alcohol [Title/Abstract])
4. (Metabolite, Alcohol [Title/Abstract]) OR (Metabolite,Gut, liver[Title/Abstract])
5. (Gut-Liver-Brain Axis [Title/Abstract]) OR (Gut-Liver Axis[Title/Abstract])
6. 6 AND 7 AND 9
7. 8 AND 10

**Sup.2. Abstracts**

| **Study** | **Country** | **Study Characteristics** | **Principal Findings** |
| --- | --- | --- | --- |
| **Llopis M et al**  **(2016)**[1] | France | Basic Study | Individual susceptibility to ALD is substantially driven by intestinal microbiota. Mice harbouring the microbiota from AH patients developed liver inflammation. |
| **Queipo-Ortuño MI et al (2012)**[2] | Spain | Clinical Study | Wine consumption can significantly modulate the growth of select gut microbiota in humans. |
| **Dubinkina VB et al (2017)**[3] | Russia | Clinical Study | There was a strong negative influence of alcohol dependence and associated liver dysfunction on gut microbiota. |
| **Clooney AG et al (2019)**[4] | Ireland | Clinical Study | The virome composition reflected alterations in bacterial composition. |
| **Koskinen K et al (2017)**[5] | Austria | Clinical Study | Similar to bacteria, human-associated archaeal communities were found to group biogeographically. |
| **Lang S et al**  **(2020)**[6] | America | Clinical Study | Patients with alcohol-associated liver disease have a lower fungal diversity with an overgrowth of Candida. |
| **Howarth DL et al (2012)**[7] | America | Basic Study | Ethanol metabolism directly impairs ER structure and function in hepatocytes. |
| **Lu R et al (2017)**[8] | America | Basic Study | Both chronic alcohol feeding and acute exposure of alcohol resulted in ISC dysregulation. |
| **Chen P et al**  **(2015)**[9] | America | Basic Study | Myosin light-chain kinase related with the intestinal barrier dysfunction and liver disease after chronic alcohol feeding |
| **Abdelmegeed MA et al**  **(2013)**[10] | America | Basic Study | CYP2E1 induced by binge alcohol seems critical in increased nitroxidative stress, gut leakage, endotoxemia, altered fat metabolism, and inflammation. |
|  |  |  |  |
| **Cho YE et al (2018)**[11] | America | Basic Study | CYP2E1, apoptosis of enterocytes, and nitration promote binge alcohol-induced gut leakiness and endotoxemia. |
| **Grander C et al (2018)**[12] | Austria | Clinical and Basic Study | Ethanol exposure diminishes intestinal A. muciniphila in humans and can be recovered by oral supplementation. |
| **Suen G et al**  **(2011)**[13] | America | Basic Study | One of the major fermentation products of Ruminococcus albus 7 is ethanol. |
| **Tsuruya A et al (2016)**[14] | Japan | Basic Study | Bacterial potentially accumulate acetaldehyde under aerobic conditions in the colon and rectum. |
| **Wang L et al**  **(2016)**[15] | America | Basic Study | REG3B or REG3G deficiency increases numbers of mucosa-associated bacteria and enhances bacteria. translocation to the mesenteric lymph nodes and liver. |
| **Nwugo CC et al (2012)**[16] | America | Basic Study | The presence of ethanol also induced the acidification of bacterial cultures and the production of indole-3-acetic acid. |
| **Chen P et al**  **(2015)**[17] | America | Basic Study | Alcohol reduces the capacity of the microbiome to synthesize saturated LCFA and the proportion of Lactobacillus species. |
| **Xie G et al**  **(2013)**[18] | America | Basic Study | Ethanol consumption altered bile acids and lipid metabolism, increased fatty acids and steroids, decreased all amino acids and branched chain amino acids |
| **Bajaj JS et al**  **(2017)**[19] | America | Clinical Study | Alcohol misuse predisposes patients to widespread dysbiosis with a toxic bile acids profile. |
| **Brandl K et al**  **(2018)**[20] | America | Clinical Study | Serum fibroblast growth factor 19 and bile acids are significantly increased in patients with alcoholic hepatitis. |
| **Kakiyama G et al (2013)**[21] | America | Clinical Study | Cirrhosis is associated with a decreased conversion of primary to secondary fecal bile acids. |
| **Ciocan D et al**  **(2018)**[22] | France | Clinical Study | Cirrhosis with severe alcoholic hepatitis show specific bile acid pool :more hydrophobic and toxic species. |
| **Kakiyama G et al (2014)**[23] | America | Clinical Study | Endotoxin, stool total bile acids, and secondary-to-primary bile acids ratios were high in current drinkers. |
| **Song Z et al (2019)**[24] | China | Clinical and Basic Study | Bile salt hydrolase-T3 found in Lactobaclillus. The phylotypes of BSH-T5 and BSH-T6 mainly from Bacteroides |
| **Chen Y et al (2011)**[13] | China | Clinical Study | Fecal microbial communities are distinct in patients with cirrhosis compared with healthy individuals. |
| **Ehlers CL et al (2012)**[25] | America | Clinical Study | Polymorphisms in ADH1B are protective against alcoholism. |
| **Miyamoto J et al (2019)**[26] | Japan | Clinical Study | Gut microbiota and host energy metabolism via the metabolites of omega-6 polyunsaturated fatty acid. |
| **Zheng TX et al (2020)**[27] | China | Basic Study | L. reuteri treatment reversed the phenotype of ethanol-induced hepatitis and metabolic disorders. |
| **Cresci GA et al (2017)**[28] | America | Basic Study | Prophylactic tributyrin supplementation mitigated effects of intestinal permeability and liver injury caused by ethanol. |
| **Roychowdhury et al (2019)**[29] | America | Basic Study | Gut dysbiosis and altered short-chain fatty acids are associated with ethanol-induced liver injury. |
| **Rios-Covian D et al (2015)**[30] | Spain | Basic Study | Enhanced formation of butyrate by F. prausnitzii in the presence of the bifidobacteria. |
| **Everard A et al (2015)**[31] | Belgium | Basic Study | A. Muciniphila increased the gut endocannabinoids that control inflammation and the gut barrier. |
| **Grander C et al (2017)**[32] | Austria | Clinical and Basic Study | Patients with alcoholic steatohepatitis showed a  significant reduction of fecal A. muc. |
| **Hendrikx T et al (2019)**[33] | America | Basic Study | Ethanol-associated dysbiosis reduces IAA and activation of the AHR to decrease expression of IL-22 in the intestine, leading to reduced expression of REG3G. |
| **Lin HR T et al (2018)**[34] | China  (Taiwan) | Basic Study | A. baumannii likely produces IAA through the indole-3-pyruvic acid (IPyA) pathway. |
| **Shu HY et al (2015)**[35] | China  (Taiwan) | Basic Study | IacR serves as a key regulator of IAA degradation in A. baumannii in the rhizosphere. |
| **Dodd D et al (2017)**[36] | America | Basic Study | Gut symbiont Clostridium sporogenes generates aromatic amino acid metabolites. |
| **Jennis M et al (2018)**[37] | America | Basic Study | IPA, a solely bacterially derived tryptophan derivative, improved intestinal barrier function in vitro and DIO mice. |
| **Mukherjee S et al (2014)**[38] | America | Basic Study | Human RegIIIa binds membrane phospholipids and kills bacteria by forming a hexameric membrane-permeabilizing oligomeric pore. |
| **Leclercq S et al (2014)**[39] | Belgium | Clinical Study | Lipopolysaccharides and peptidoglycans stimulate specific inflammatory pathways in peripheral blood mononuclear cells that are correlated with alcohol craving. |
| **Yoon SI et al (2012)**[40] | America | Basic Study | TLR5 binding to bacterial flagellin activates signaling through the transcription factor NF-kB and triggers an innate immune response to the invading pathogen. |
| **Duan Y et al (2019)**[41] | America | Clinical Study | Cytolytic E. faecalis related with severe clinical outcomes and increased mortality in patients with alcoholic hepatitis. |
| **Yang AM et al (2017)**[42] | America | Clinical and Basic Study | Alcoholic cirrhosis patients had increased systemic exposure and immune response to mycobiota. |
| **Hsu TC et al (2017)**[43] | China  (Taiwan) | Basic Study | Supplementary GMNL-32, GMNL-89 or GMNL-263 in mice ameliorates hepatic apoptosis and inflammation. |
| **Wang Y et al (2013)**[44] | America | Basic Study | Probiotic LGG treatment reduced alcohol-induced hepatic inflammation by attenuation of TNFa production. |
| **Seo B et al (2020)**[45] | Korea | Basic Study | The flagellin of R. intestinalis, possibly through Toll-like receptor 5 recognition, recovers gut barrier integrity. |
| **Païssé S et al (2016)**[46] | France | Clinical Study | A diversified microbiome exists in healthy blood. |
| **Puri P et al (2018)**[47] | America | Clinical Study | Heavy alcohol consumption appears to be the primary driver of changes in the circulating microbiome. |
| **Beisel C et al (2016)**[48] | Germany | Clinical Study | The high rate of Enterococcus infections suggests that commonly used antibiotics, such as cephalosporins. |
| **Bajaj JS et al**  **(2015)**[49] | America | Clinical Study | Patients with cirrhosis have impaired salivary defenses, salivary dysbiosis and worse inflammation. |
| **Bajaj JS et al**  **(2018)**[50] | America | Clinical Study | Systematic periodontal therapy improved modulated salivary and stool microbial dysbiosis. |
| **Mutlu EA et al (2012)**[51] | America | Clinical Study | Bacterial microbiome is altered in alcoholics. The change is persistent and correlates with endotoxemia. |
| **Leclercq S et al (2014)**[52] | Belgium | Clinical Study | Alcohol-dependent subjects developed gut leakiness, associated with depression, anxiety, and alcohol craving. |
| **Williams BB et al (2014)**[53] | America | Basic Study | Two enzymes can decarboxylate tryptophan to form the  β-arylamine neurotransmitter tryptamine. |
| **Enoch MA et al (2011)**[54] | America | Clinical Study | Increased synaptic 5-HT coupled with 5-HT 3 receptor responsiveness may result in dopamine transmission. |
| **Mao YK et al (2013)**[55] | Canada | Basic Study | polysaccharide A of bacteria is necessary and sufficient for the neuronal effects. |
| **Pokusaeva K et al (2017)**[56] | America | Basic Study | Targeting GABAergic signals along microbiome-gut-brain axis represents a new treatment for abdominal pain. |
| **Tsai YS et al (2020)**[57] | China  (Taiwan) | Basic Study | A decrease in the serum ALT, liver TG, and liver TC levels in the GKS6, GKM3, and GKLC1 groups |
| **Zhao W et al (2020)**[58] | China | Basic Study | Fecal microbiota from patients with alcoholism did induce a status like alcohol dependence in C57BL/6J mice. |
| **Kelly JR et al (2016)**[59] | Ireland | Basic Study | Gut microbiota may play a causal role in the development of features of depression |
| **Xu Z et al (2019)**[60] | China | Clinical Study | Gut dysbiosis was correlated with alcohol-induced neuropsychic behaviors and BDNF/Gabra1 expression. |
| **Bahorik A et al (2021)**[61] | America | Clinical Study | Alcohol use disorder appears to be associated with a more than threefold increase of dementia. |
| **Wang X et al (2021)**[62] | China | Basic Study | Gut dysbiosis-promoted neuroinflammation in AD progression. |
| **Riggio O et al (2010)**[63] | Italy | Basic Study | Indole correlates with HE and has a significant intestinal production and hepatic extraction |
| **Sung CM et al (2019)**[64] | China  (Taiwan) | Clinical Study | Acute HE-specific gut OTUs were identified that may be involved in HE development and clinical outcomes. |
| **Bajaj JS et al**  **(2014)**[65] | America | Clinical Study | Progressive changes in the gut microbiome accompany cirrhosis and become more severe during decompensation. |
| **Lages EJ et al**  **(2015)**[66] | Brazil | Clinical Study | The worse periodontal status and the higher levels of P. intermedia, E. corrodens, F. nucleatum, and IL-1β. |
| **Vlachogiannakos J et al**  **(2013)**[67] | Greece | Clinical Study | Long-term rifaximin administration is associated with reduced risk of developing complications of portal hypertension and improved survival. |
| **Philips CA et al (2018)**[68] | India | Clinical Study | Healthy donor fecal transplantation for severe alcoholic hepatitis improves survival |

**Sup 3. Details of the gut-brain cross talk in alcohol withdrawal reaction**

Chronic alcohol consumption may impact inflammation directly at the brain level and in the periphery. Alcohol induces intestinal permeability and microbial dysbiosis that contribute to the inflammatory cascade. Damage to the intestinal epithelial layer causes the leakage of bacterial products such as lipopolysaccharide (LPS) that can enter the blood and reach the liver. In response, immune cells secrete cytokines which are transported via the blood stream to the brain causing neural damage that leads to cognitive and emotional impairments and sensitisation of stress-response pathways (hypothalamic pituitary adrenal [HPA] axis). Alcohol-derived alterations in stress and inflammatory responses may contribute to fronto-limbic alterations, resulting in emotional dysregulation[69].

In the addiction circle, at the binge/intoxication stage, alcohol use is mainly motivated by positive reinforcement. Repeated intoxications might initiate microbiome neuro-immune-affective imbalances that progressively feeds allostasis, contributing to alterations in frontolimbic networks and early emotional dysregulation[70, 71]. At the withdrawal/negative affect stage, both negative affect related to acute withdrawal and lasting emotional disturbances may drive negative reinforcement processes that might be further exaggerated by microbiome-immune interdependencies[72, 73]. At the preoccupation/anticipation stage, impairments in top-down connectivity result in weakened affective control, with relevant implications for stress-induced craving and relapse[74]. Underlying alcohol-related microbiome alterations might, therefore, contribute to a vicious circle of emotional dysregulation that is likely to accelerate the transition to compulsive alcohol use.

Microbiome-immune disruptions has been identified as potential mediators of fronto-limbic anomalies and derived emotional dysregulation in the alcohol addiction cycle. Starting with the gut, alcohol withdrawal may lead to a decrease in the protective colonies (like Bacteroidetes species) or an increase in the pathogenic colonies (like Proteobacteria species, Actinobacteria species), both of which can drive changes in inflammatory signaling and cytokine release (like TNFα, LPS, IL-1β, IL-6, IL-8, and IL-10)[70]. On the one hand, a “leaky gut” permits inflammatory molecules to leak outside of the gut into the bloodstream or surrounding adipocytes. Once in the bloodstream, even if the blood-brain barrier is intact, peripheral cytokines have the potential to enter the brain via active transport and potentially induce their own synthesis. Endotoxin-induced peripheral inflammation can activate microglia and cause neuroinflammatory responses[75, 76]. On the other hand, such alterations may prime the vagus nerve to withdraw or alter its neuroprotective afferent signals or promote vagal signals that are in some way harmful[69]. With the brain primed by vagal afferent alterations, it may be more susceptible to influence from the peripheral inflammatory response that occurs in response to alcohol withdrawal. Before that, we need to know that the vagus afferent nerve senses change in relative bacterial abundance through two pathways: one route of communication may be through direct interactions between vagal afferent neurons and bacteria or their metabolic products[77]. Another route includes the effects of paracrine signaling from enterochromaffin (EC) cells and from enteroendocrine (EE) cells that interact with the gut lumen causing secretion of factors that affect nearby cells in the gut wall[78]. Neuroinflammation in regions like the central nucleus of the amygdala (CeA) and others can contribute to emotional dysregulation seen in withdrawal. The state of the gut microbiome is reported to the brain via vagal projections to the nucleus tractus solitarius (NTS) by changes in the activity of the vagus nerve and in the neurochemical phenotype of the vagal afferent neurons in the nodose ganglia and their targets in the NTS. The NTS in turn has viscerosensory projections to the central nucleus of the amygdala (CeA) that involves norepinephrine (NE)[79]. Decreased activity of these projections would lead to a loss of its anti-inflammatory activity. For example, neurons expressing glucagon-like peptide 1 (GLP-1) and glutamate have been described to participate in these NTS viscerosensory projections to the CeA in a proposed anxiogenic GLP-1 pathway to the corticotropin-releasing factor (CRF) neurons in the CeA[80, 81]. The CeA contains a dense population of CRF neurons that co-express GABA and a dense plexus of CRF terminals[79]. CRF is a key neuropeptide mediator of stress and neuroinflammatory responses linked to withdrawal. Thus increased activity of the NTS GLP-1 neurons by vagal inputs may well be pro-inflammatory and anxiogenic in the CeA mediating stress and anxiety.

**Reference：**

1. Llopis M, Cassard AM, Wrzosek L, et al. Intestinal microbiota contributes to individual susceptibility to alcoholic liver disease. *Gut*. 2016;65:830-839.

2. Queipo-Ortuño MI, Boto-Ordóñez M, Murri M, et al. Influence of red wine polyphenols and ethanol on the gut microbiota ecology and biochemical biomarkers. *Am J Clin Nutr*. 2012;95:1323-1334.

3. Dubinkina VB, Tyakht AV, Odintsova VY, et al. Links of gut microbiota composition with alcohol dependence syndrome and alcoholic liver disease. *Microbiome*. 2017;5:141.

4. Clooney AG, Sutton TDS, Shkoporov AN, et al. Whole-Virome Analysis Sheds Light on Viral Dark Matter in Inflammatory Bowel Disease. *Cell Host Microbe*. 2019;26:764-778.e765.

5. Koskinen K, Pausan MR, Perras AK, et al. First Insights into the Diverse Human Archaeome: Specific Detection of Archaea in the Gastrointestinal Tract, Lung, and Nose and on Skin. *mBio*. 2017;8.

6. Lang S, Duan Y, Liu J, et al. Intestinal Fungal Dysbiosis and Systemic Immune Response to Fungi in Patients With Alcoholic Hepatitis. *Hepatology*. 2020;71:522-538.

7. Howarth DL, Vacaru AM, Tsedensodnom O, et al. Alcohol disrupts endoplasmic reticulum function and protein secretion in hepatocytes. *Alcohol Clin Exp Res*. 2012;36:14-23.

8. Lu R, Voigt RM, Zhang Y, et al. Alcohol Injury Damages Intestinal Stem Cells. *Alcohol Clin Exp Res*. 2017;41:727-734.

9. Chen P, Stärkel P, Turner JR, et al. Dysbiosis-induced intestinal inflammation activates tumor necrosis factor receptor I and mediates alcoholic liver disease in mice. *Hepatology*. 2015;61:883-894.

10. Abdelmegeed MA, Banerjee A, Jang S, et al. CYP2E1 potentiates binge alcohol-induced gut leakiness, steatohepatitis, and apoptosis. *Free Radic Biol Med*. 2013;65:1238-1245.

11. Cho YE, Yu LR, Abdelmegeed MA, et al. Apoptosis of enterocytes and nitration of junctional complex proteins promote alcohol-induced gut leakiness and liver injury. *J Hepatol*. 2018;69:142-153.

12. Grander C, Adolph TE, Wieser V, et al. Recovery of ethanol-induced Akkermansia muciniphila depletion ameliorates alcoholic liver disease. *Gut*. 2018;67:891-901.

13. Suen G, Stevenson DM, Bruce DC, et al. Complete genome of the cellulolytic ruminal bacterium Ruminococcus albus 7. *J Bacteriol*. 2011;193:5574-5575.

14. Tsuruya A, Kuwahara A, Saito Y, et al. Major Anaerobic Bacteria Responsible for the Production of Carcinogenic Acetaldehyde from Ethanol in the Colon and Rectum. *Alcohol Alcohol*. 2016;51:395-401.

15. Wang L, Fouts DE, Stärkel P, et al. Intestinal REG3 Lectins Protect against Alcoholic Steatohepatitis by Reducing Mucosa-Associated Microbiota and Preventing Bacterial Translocation. *Cell Host Microbe*. 2016;19:227-239.

16. Nwugo CC, Arivett BA, Zimbler DL, et al. Effect of ethanol on differential protein production and expression of potential virulence functions in the opportunistic pathogen Acinetobacter baumannii. *PLoS One*. 2012;7:e51936.

17. Chen P, Miyamoto Y, Mazagova M, et al. Microbiota Protects Mice Against Acute Alcohol-Induced Liver Injury. *Alcohol Clin Exp Res*. 2015;39:2313-2323.

18. Xie G, Zhong W, Li H, et al. Alteration of bile acid metabolism in the rat induced by chronic ethanol consumption. *Faseb j*. 2013;27:3583-3593.

19. Bajaj JS, Kakiyama G, Zhao D, et al. Continued Alcohol Misuse in Human Cirrhosis is Associated with an Impaired Gut-Liver Axis. *Alcohol Clin Exp Res*. 2017;41:1857-1865.

20. Brandl K, Hartmann P, Jih LJ, et al. Dysregulation of serum bile acids and FGF19 in alcoholic hepatitis. *J Hepatol*. 2018;69:396-405.

21. Kakiyama G, Pandak WM, Gillevet PM, et al. Modulation of the fecal bile acid profile by gut microbiota in cirrhosis. *J Hepatol*. 2013;58:949-955.

22. Ciocan D, Voican CS, Wrzosek L, et al. Bile acid homeostasis and intestinal dysbiosis in alcoholic hepatitis. *Aliment Pharmacol Ther*. 2018;48:961-974.

23. Kakiyama G, Hylemon PB, Zhou H, et al. Colonic inflammation and secondary bile acids in alcoholic cirrhosis. *Am J Physiol Gastrointest Liver Physiol*. 2014;306:G929-937.

24. Song Z, Cai Y, Lao X, et al. Taxonomic profiling and populational patterns of bacterial bile salt hydrolase (BSH) genes based on worldwide human gut microbiome. *Microbiome*. 2019;7:9.

25. Chen Y, Yang F, Lu H, et al. Characterization of fecal microbial communities in patients with liver cirrhosis. *Hepatology*. 2011;54:562-572.

26. Ehlers CL, Liang T and Gizer IR. ADH and ALDH polymorphisms and alcohol dependence in Mexican and Native Americans. *Am J Drug Alcohol Abuse*. 2012;38:389-394.

27. Miyamoto J, Igarashi M, Watanabe K, et al. Gut microbiota confers host resistance to obesity by metabolizing dietary polyunsaturated fatty acids. *Nat Commun*. 2019;10:4007.

28. Zheng TX, Pu SL, Tan P, et al. Liver Metabolomics Reveals the Effect of Lactobacillus reuteri on Alcoholic Liver Disease. *Front Physiol*. 2020;11:595382.

29. Cresci GA, Glueck B, McMullen MR, et al. Prophylactic tributyrin treatment mitigates chronic-binge ethanol-induced intestinal barrier and liver injury. *J Gastroenterol Hepatol*. 2017;32:1587-1597.

30. Roychowdhury S, Glueck B, Han Y, et al. A Designer Synbiotic Attenuates Chronic-Binge Ethanol-Induced Gut-Liver Injury in Mice. *Nutrients*. 2019;11.

31. Rios-Covian D, Gueimonde M, Duncan SH, et al. Enhanced butyrate formation by cross-feeding between Faecalibacterium prausnitzii and Bifidobacterium adolescentis. *FEMS Microbiol Lett*. 2015;362.

32. Everard A, Belzer C, Geurts L, et al. Cross-talk between Akkermansia muciniphila and intestinal epithelium controls diet-induced obesity. *Proc Natl Acad Sci U S A*. 2013;110:9066-9071.

33. Grander C GF, Adolph TE, et al. Ethanol-induced depletion of Akkermansia muciniphila drives alcoholic liver disease. *Journal of Hepatology*.2017;66(1):S347.

34. Hendrikx T, Duan Y, Wang Y, et al. Bacteria engineered to produce IL-22 in intestine induce expression of REG3G to reduce ethanol-induced liver disease in mice. *Gut*. 2019;68:1504-1515.

35. Lin HR, Shu HY and Lin GH. Biological roles of indole-3-acetic acid in Acinetobacter baumannii. *Microbiol Res*. 2018;216:30-39.

36. Shu HY, Lin LC, Lin TK, et al. Transcriptional regulation of the iac locus from Acinetobacter baumannii by the phytohormone indole-3-acetic acid. *Antonie Van Leeuwenhoek*. 2015;107:1237-1247.

37. Dodd D, Spitzer MH, Van Treuren W, et al. A gut bacterial pathway metabolizes aromatic amino acids into nine circulating metabolites. *Nature*. 2017;551:648-652.

38. Jennis M, Cavanaugh CR, Leo GC, et al. Microbiota-derived tryptophan indoles increase after gastric bypass surgery and reduce intestinal permeability in vitro and in vivo. *Neurogastroenterol Motil*. 2018;30.

39. Mukherjee S, Zheng H, Derebe MG, et al. Antibacterial membrane attack by a pore-forming intestinal C-type lectin. *Nature*. 2014;505:103-107.

40. Leclercq S, Matamoros S, Cani PD, et al. Intestinal permeability, gut-bacterial dysbiosis, and behavioral markers of alcohol-dependence severity. *Proc Natl Acad Sci U S A*. 2014;111:E4485-4493.

41. Yoon SI, Kurnasov O, Natarajan V, et al. Structural basis of TLR5-flagellin recognition and signaling. *Science*. 2012;335:859-864.

42. Duan Y, Llorente C, Lang S, et al. Bacteriophage targeting of gut bacterium attenuates alcoholic liver disease. *Nature*. 2019;575:505-511.

43. Yang AM, Inamine T, Hochrath K, et al. Intestinal fungi contribute to development of alcoholic liver disease. *J Clin Invest*. 2017;127:2829-2841.

44. Hsu TC, Huang CY, Liu CH, et al. Lactobacillus paracasei GMNL-32, Lactobacillus reuteri GMNL-89 and L. reuteri GMNL-263 ameliorate hepatic injuries in lupus-prone mice. *Br J Nutr*. 2017;117:1066-1074.

45. Wang Y, Liu Y, Kirpich I, et al. Lactobacillus rhamnosus GG reduces hepatic TNFα production and inflammation in chronic alcohol-induced liver injury. *J Nutr Biochem*. 2013;24:1609-1615.

46. Seo B, Jeon K, Moon S, et al. Roseburia spp. Abundance Associates with Alcohol Consumption in Humans and Its Administration Ameliorates Alcoholic Fatty Liver in Mice. *Cell Host Microbe*. 2020;27:25-40.e26.

47. Païssé S, Valle C, Servant F, et al. Comprehensive description of blood microbiome from healthy donors assessed by 16S targeted metagenomic sequencing. *Transfusion*. 2016;56:1138-1147.

48. Puri P, Liangpunsakul S, Christensen JE, et al. The circulating microbiome signature and inferred functional metagenomics in alcoholic hepatitis. *Hepatology*. 2018;67:1284-1302.

49. Beisel C, Blessin U, Schulze Zur Wiesch J, et al. Infections complicating severe alcoholic hepatitis: Enterococcus species represent the most frequently identified pathogen. *Scand J Gastroenterol*. 2016;51:807-813.

50. Bajaj JS, Betrapally NS, Hylemon PB, et al. Salivary microbiota reflects changes in gut microbiota in cirrhosis with hepatic encephalopathy. *Hepatology*. 2015;62:1260-1271.

51. Bajaj JS, Matin P, White MB, et al. Periodontal therapy favorably modulates the oral-gut-hepatic axis in cirrhosis. *Am J Physiol Gastrointest Liver Physiol*. 2018;315:G824-g837.

52. Mutlu EA, Gillevet PM, Rangwala H, et al. Colonic microbiome is altered in alcoholism. *Am J Physiol Gastrointest Liver Physiol*. 2012;302:G966-978.

53. Leclercq S, De Saeger C, Delzenne N, et al. Role of inflammatory pathways, blood mononuclear cells, and gut-derived bacterial products in alcohol dependence. *Biol Psychiatry*. 2014;76:725-733.

54. Williams BB, Van Benschoten AH, Cimermancic P, et al. Discovery and characterization of gut microbiota decarboxylases that can produce the neurotransmitter tryptamine. *Cell Host Microbe*. 2014;16:495-503.

55. Enoch MA, Gorodetsky E, Hodgkinson C, et al. Functional genetic variants that increase synaptic serotonin and 5-HT3 receptor sensitivity predict alcohol and drug dependence. *Mol Psychiatry*. 2011;16:1139-1146.

56. Mao YK, Kasper DL, Wang B, et al. Bacteroides fragilis polysaccharide A is necessary and sufficient for acute activation of intestinal sensory neurons. *Nat Commun*. 2013;4:1465.

57. Pokusaeva K, Johnson C, Luk B, et al. GABA-producing Bifidobacterium dentium modulates visceral sensitivity in the intestine. *Neurogastroenterol Motil*. 2017;29.

58. Tsai YS, Lin SW, Chen YL, et al. Effect of probiotics Lactobacillus paracasei GKS6, L. plantarum GKM3, and L. rhamnosus GKLC1 on alleviating alcohol-induced alcoholic liver disease in a mouse model. *Nutr Res Pract*. 2020;14:299-308.

59. Kelly JR, Borre Y, C OB, et al. Transferring the blues: Depression-associated gut microbiota induces neurobehavioural changes in the rat. *J Psychiatr Res*. 2016;82:109-118.

60. Xu Z, Wang C, Dong X, et al. Chronic alcohol exposure induced gut microbiota dysbiosis and its correlations with neuropsychic behaviors and brain BDNF/Gabra1 changes in mice. *Biofactors*. 2019;45:187-199.

61. Bahorik A, Bobrow K, Hoang T, et al. Increased risk of dementia in older female US veterans with alcohol use disorder. *Addiction*. 2021;116:2049-2055.

62. Wang X, Sun G, Feng T, et al. Sodium oligomannate therapeutically remodels gut microbiota and suppresses gut bacterial amino acids-shaped neuroinflammation to inhibit Alzheimer's disease progression. *Cell Res*. 2019;29:787-803.

63. Riggio O, Mannaioni G, Ridola L, et al. Peripheral and splanchnic indole and oxindole levels in cirrhotic patients: a study on the pathophysiology of hepatic encephalopathy. *Am J Gastroenterol*. 2010;105:1374-1381.

64. Sung CM, Lin YF, Chen KF, et al. Predicting Clinical Outcomes of Cirrhosis Patients With Hepatic Encephalopathy From the Fecal Microbiome. *Cell Mol Gastroenterol Hepatol*. 2019;8:301-318.e302.

65. Bajaj JS, Heuman DM, Hylemon PB, et al. Altered profile of human gut microbiome is associated with cirrhosis and its complications. *J Hepatol*. 2014;60:940-947.

66. Lages EJ, Costa FO, Cortelli SC, et al. Alcohol Consumption and Periodontitis: Quantification of Periodontal Pathogens and Cytokines. *J Periodontol*. 2015;86:1058-1068.

67. Vlachogiannakos J, Viazis N, Vasianopoulou P, et al. Long-term administration of rifaximin improves the prognosis of patients with decompensated alcoholic cirrhosis. *J Gastroenterol Hepatol*. 2013;28:450-455.

68. Philips CA, Phadke N, Ganesan K, et al. Corticosteroids, nutrition, pentoxifylline, or fecal microbiota transplantation for severe alcoholic hepatitis. *Indian J Gastroenterol*. 2018;37:215-225.

69. Carbia C, Lannoy S, Maurage P, et al. A biological framework for emotional dysregulation in alcohol misuse: from gut to brain. *Mol Psychiatry*. 2021;26:1098-1118.

70. Gorky J and Schwaber J. The role of the gut-brain axis in alcohol use disorders. *Prog Neuropsychopharmacol Biol Psychiatry*. 2016;65:234-241.

71. Koob GF and Volkow ND. Neurobiology of addiction: a neurocircuitry analysis. *Lancet Psychiatry*. 2016;3:760-773.

72. Koob GF and Schulkin J. Addiction and stress: An allostatic view. *Neurosci Biobehav Rev*. 2019;106:245-262.

73. Koob G and Kreek MJ. Stress, dysregulation of drug reward pathways, and the transition to drug dependence. *Am J Psychiatry*. 2007;164:1149-1159.

74. Volkow ND, Koob GF and McLellan AT. Neurobiologic Advances from the Brain Disease Model of Addiction. *N Engl J Med*. 2016;374:363-371.

75. Skinner RA, Gibson RM, Rothwell NJ, et al. Transport of interleukin-1 across cerebromicrovascular endothelial cells. *Br J Pharmacol.* 2009;156:1115-1123.

76. Keita AV and Söderholm JD. The intestinal barrier and its regulation by neuroimmune factors. *Neurogastroenterol Motil*. 2010;22:718-733.

77. Mayer EA. Gut feelings: the emerging biology of gut-brain communication. *Nat Rev Neurosci*. 2011;12:453-466.

78. Rhee SH, Pothoulakis C and Mayer EA. Principles and clinical implications of the brain-gut-enteric microbiota axis. *Nat Rev Gastroenterol Hepatol*. 2009;6:306-314.

79. Dockray GJ. Gastrointestinal hormones and the dialogue between gut and brain. *J Physiol*. 2014;592:2927-2941.

80. Maniscalco JW, Kreisler AD and Rinaman L. Satiation and stress-induced hypophagia: examining the role of hindbrain neurons expressing prolactin-releasing Peptide or glucagon-like Peptide 1. *Front Neurosci*. 2012;6:199.

81. Zheng H, Cai L and Rinaman L. Distribution of glucagon-like peptide 1-immunopositive neurons in human caudal medulla. *Brain Struct Funct*. 2015;220:1213-1219.
